# A machine learning approach with SHAP interpretability for classifying drug craving levels

**DOI:** 10.3389/fpubh.2026.1752380

**Published:** 2026-05-11

**Authors:** Weiqi Zeng, Fang Liu, Ting Liang, Cui Zhao, Xiaoting Liu, Ping Yang

**Affiliations:** 1 Clinical Medical College, Hunan University of Chinese Medicine, Changsha, Hunan, China; 2 The Second People’s Hospital of Hunan Province (Brain Hospital of Hunan Province), Changsha, Hunan, China; 3 College of Traditional Chinese Medicine, Hunan University of Chinese Medicine, Changsha, Hunan, China

**Keywords:** drug craving, feature importance, logistic regression, machine learning, SHAP

## Abstract

**Background:**

Drug addiction is a chronic relapsing brain disease. Drug craving is the strongest independent predictor of relapse. However, traditional linear models often fail to capture complex non-linear addiction patterns. Although machine learning (ML) demonstrates superior performance, its “black-box” nature limits clinical credibility.

**Objective:**

This study aimed to construct a classification model for drug craving scores based on ML and explore the impact of multifactorial features.

**Methods:**

A total of 692 abstainers were recruited from Compulsory Isolation Drug Rehabilitation Centers. Craving was assessed using a 34-item Drug Craving Scale. After preprocessing (imputing missing values, removing outliers via IQR), 629 valid samples remained. Eighteen demographic and behavioral features were analyzed. Samples were split 7:3 into training and test sets. SMOTE was employed for class imbalance. Seven algorithms—including Logistic Regression, XGBoost, and LightGBM—were compared. The optimal model was selected using 10-fold cross-validation and grid search, then evaluated on the independent test set using multi-dimensional metrics. SHAP was introduced for interpretability.

**Results:**

Logistic Regression was the optimal model. On the independent test set, it achieved 66.13% accuracy and 0.85 micro-average AUC, demonstrating encouraging potential in identifying the high-craving group (AUC = 0.84). SHAP quantified feature contributions: frequency of drug use, duration of use, and heroin use were core factors. Behavioral features positively correlated with high craving, whereas sociodemographic features exhibited a protective effect that diminished as addiction severity increased.

**Conclusion:**

The Logistic Regression model combines predictive performance and interpretability. By applying SHAP, this study visually elucidated specific feature contributions, enhancing model transparency for preliminary clinical evaluations, pending rigorous validation in independent and diverse populations.

## 1 Introduction

Drug abuse and addiction, characterized as a global chronic relapsing brain disease, have become major public health issues that seriously threaten human health and social security (1). According to the World Drug Report 2024 released by the United Nations Office on Drugs and Crime (UNODC), approximately 292 million people worldwide have used drugs, representing a 20% increase from a decade ago. Among them, more than 64 million people suffer from Drug Use Disorders (2). Although existing interventions—such as compulsory isolation for detoxification, community-based rehabilitation, and opioid substitution treatment—can assist addicts in achieving physiological detoxification, relapse rates remain persistently high after individuals return to society due to neuroadaptive changes and the induction of environmental cues (3). Previous studies indicate that the relapse rate for individuals with substance use disorders reaches as high as 60% within the first year of abstinence, and some individuals may still relapse even after decades of abstinence (4). Therefore, accurately identifying high-risk groups for relapse and implementing effective interventions have become the most significant challenges in the field of addiction medicine.

Within this complex pathological process, drug craving is defined as an intense, uncontrollable psychological desire and compulsive urge for addictive substances. This pathological psychological state has been established as a core diagnostic criterion in the Diagnostic and Statistical Manual of Mental Disorders, Fifth Edition (DSM-5) (5, 6). A large-scale meta-analysis published in JAMA Psychiatry by Vafaie and Kober (5) further confirmed that craving intensity is the strongest independent predictor of post-abstinence relapse, with predictive validity significantly superior to demographic characteristics and basic drug use history. Previous neurobiological studies have indicated that the emergence of craving is closely associated with dysfunction of the mesolimbic dopamine system (7). Chronic drug exposure leads to the downregulation of dopamine D2 receptors, inhibition of prefrontal cortex function, and abnormal amygdala-prefrontal connectivity. These neuroadaptive changes render individuals hypersensitive to drug-related cues, thereby triggering intense impulses for relapse (8). Furthermore, Volkow et al. (9) confirmed through Positron Emission Tomography (PET) studies that chronic drug use results in reduced dopamine D2 receptor availability, which attenuates responsiveness to natural rewards and exacerbates pathological craving for drugs.

However, despite the pivotal role of craving in relapse prediction, existing assessment and prediction methods face numerous limitations. Historically, clinical assessment of craving has relied primarily on Visual Analog Scales (VAS) and various self-report questionnaires. These traditional methods are not only susceptible to cognitive biases, emotional states, and social desirability effects of the subjects but, more importantly, are often based on linear assumptions, failing to adequately capture the latent interactive patterns of multi-dimensional features underlying the complex system of addiction (10). Recent research on the “Incubation of Craving” indicates that during abstinence, craving levels exhibit complex dynamic changes rather than a simple linear decline over time (11). Furthermore, there exists a complex coupling relationship between individual psychosocial characteristics (e.g., chronic stress, social isolation) and biological mechanisms (e.g., HPA axis activation, neural synaptic plasticity) (12). The revised model of addiction as a brain disease proposed by Heilig et al. (13) particularly emphasizes the biological embedding of social factors, suggesting that the “double hit” of environmental stress and neural vulnerability is key to the maintenance of craving. Faced with this complex bio-psycho-social interaction network, traditional univariate analyses or linear regression models often encounter bottlenecks in prediction accuracy due to their inability to process high-dimensional heterogeneous data, thus failing to fully meet the demands of precision clinical diagnosis and treatment.

With the rise of computational psychiatry, artificial intelligence technologies, represented by Machine Learning (ML), have provided new opportunities to address these challenges. Unlike traditional statistical methods, machine learning algorithms are capable of automatically capturing complex patterns within high-dimensional data, thereby uncovering predictive regularities that are difficult to detect through manual hypotheses (14, 15). Whether identifying neuropathological features in neuroimaging data or predicting relapse risk based on behavioral data, machine learning has demonstrated potential superior to traditional models in handling the high complexity and heterogeneity of addictive behaviors (16). However, as the complexity of machine learning algorithms increases, the “black-box” nature of these models becomes increasingly pronounced. While complex models can yield high-precision prediction results, clinicians are often unable to ascertain the rationale behind their decisions. This opacity in the decision-making process limits the credibility of machine learning models in clinical practice to a certain extent, hindering their translation into substantive evidence for clinical intervention (17).

To overcome the bottlenecks in clinical application caused by the black-box nature of complex models, this study introduced the SHAP (SHapley Additive exPlanations) post-hoc explanation framework (18). This method ingeniously applies classical game theory concepts to machine learning, with its core logic treating the prediction model as a complex game system and the input features as players participating in the game. By calculating the average marginal contribution of each feature to the prediction outcome under different feature combinations, SHAP is able to assign a unique Shapley value satisfying the additivity property to each feature of every sample. Compared to traditional methods, SHAP not only ensures the consistency of feature importance evaluation but also effectively identifies specific associations between variables, thereby providing reliable methodological support for the in-depth analysis of biomedical data (19).

In summary, this study aims to construct a machine learning-based classification prediction model for drug craving to address the limitations of existing linear models in handling complex data. We included abstainers from Compulsory Isolation Drug Rehabilitation Centers in Hunan Province and collected multi-dimensional data, comprising demographic characteristics and drug-use behavioral features. Addressing the class imbalance identified in the data, we employed the Synthetic Minority Over-sampling Technique (SMOTE) for balancing. Subsequently, seven machine learning models with distinct underlying principles were constructed, including Logistic Regression, eXtreme Gradient Boosting (XGBoost), Light Gradient Boosting Machine (LightGBM), Decision Tree, Gradient Boosting, Gaussian Naive Bayes (GaussianNB), and K-Nearest Neighbors (KNN). We performed a comparative evaluation of these seven algorithms on the training set using 10-fold cross-validation based on metrics such as Accuracy, F1-score, and Area Under the Receiver Operating Characteristic Curve (AUC) to select the optimal model. Furthermore, the generalization performance of the optimal model and its discriminative capability for different craving categories were evaluated on an independent test set using Macro-Average and Micro-Average ROC curves and confusion matrices. On this basis, the SHAP method was applied to elucidate the decision-making process of the optimal model. The core objective of this study is not merely to provide an auxiliary tool with robust predictive efficacy but also to offer a supplementary perspective grounded in computational psychiatry for understanding factors influencing drug craving by demonstrating the model’s integration of multi-dimensional features.

## 2 Materials and methods

### 2.1 Data and participants

This study was approved by the Ethics Committee of the Second People’s Hospital of Hunan Province (Ethics Approval No.: 2022045), and all research procedures were conducted in strict accordance with the ethical guidelines of the Declaration of Helsinki. Data collection took place from March 2023 to July 2023. A total of 692 drug users were recruited from Compulsory Isolation Drug Rehabilitation Centers in Hunan Province using a questionnaire survey method. Participants were screened based on the following inclusion criteria: (1) meeting the diagnostic criteria for Substance-Related and Addictive Disorders as defined by the Diagnostic and Statistical Manual of Mental Disorders, Fifth Edition (DSM-5); (2) age ≥18 years; (3) male gender; (4) ability to comprehend the questionnaire items and provision of written informed consent. Exclusion criteria were: (1) presence of schizophrenia, bipolar disorder, or major depressive disorder as defined by the DSM-5; (2) refusal to participate in the questionnaire survey. All data were collected using standardized scales administered by uniformly trained research assistants through face-to-face interviews, with each participant requiring approximately 30–40 min to complete the assessment.

### 2.2 Study variables

#### 2.2.1 Predictor variables

A self-designed General Information Scale was employed to collect 22 potential population-related factors (Supplementary Table 1), comprising demographic and drug-related characteristics. Demographic characteristics included Name Initials, Gender, Age, Date of Birth, Education Level, Marital Status, Occupation, Monthly household income per capita, and Family atmosphere. Drug-related characteristics included Date of Admission, Expected Release Date, Types of drugs used (e.g., Methamphetamine, MDMA, Amphetamines, Ketamine, Heroin), primary method of drug use (e.g., Snorting/Smoking, Injection, Oral ingestion), Years of drug use, average daily amount used, and frequency of use. Prior to model construction, preprocessing and feature engineering were performed on selected raw variables. Specifically: (1) the “Name Initials” feature was removed due to its lack of relevance for modeling; (2) the “Date of Birth” variable was excluded due to its redundancy with “Age,” with “Age” being directly input into the model as a demographic feature; (3) “Duration of detention (days)” was derived from “Date of Admission” and “Expected Release Date” and included as a new continuous feature; and (4) specific drug types (e.g., Methamphetamine, MDMA, Amphetamines (Speed), Ketamine, Heroin) were processed as binary features, coded as “1” for use and “0” for non-use.

#### 2.2.2 Outcome variables

We assessed craving levels using a self-developed Self-rating Drug Craving Scale (see Supplementary Table 1 for details). Developed based on theories of classical and operant conditioning, the scale comprises 34 items covering five dimensions of drug craving: (1) Reward-based drug craving; (2) Reflexive drug craving; (3) Social drug craving; (4) Negative drug craving; and (5) Relief-oriented drug craving. Participants were asked to rate each item on a scale from 1 (Strongly Disagree) to 7 (Strongly Agree) based on their actual conditions. The total score was obtained by summing the scores of the 34 items, with a theoretical range of 34–238. This study aimed to establish an exploratory classification system with potential clinical utility. Pending external validation, the current internally defined thresholds serve as a preliminary framework. Given the lack of generally accepted standards for classifying the total score of this scale into “low,” “moderate,” and “high” categories, we referenced the grading method of the Visual Analogue Scale (VAS) (20), a widely recognized effective tool in craving assessment. The continuous total score was divided into three approximate intervals to classify craving levels into three categories: (1) Low Craving (total score 0–102); (2) Moderate Craving (103–170); and (3) High Craving (171–238). To evaluate the credibility of this classification method, two statistical analyses were performed. First, the Cronbach’s *α* coefficient of the total scale was calculated to assess the internal consistency of items in measuring the same construct, thereby testing the internal reliability of the scale. Second, the Kappa coefficient was calculated to compare the classification results based on the total score in this study with the VAS grading, in order to test the logical consistency between the cut-off value settings of this study and the VAS grading.

### 2.3 Statistical analysis

#### 2.3.1 Data preprocessing

In the initial stage of the study, raw data from 692 samples were collected and preprocessed. First, missing values were imputed: continuous variables were filled using the mean, while categorical variables were filled using the mode. Subsequently, the Interquartile Range (IQR) rule was applied to identify and handle outliers potentially caused by anomalies or extreme deviations. Specifically, samples falling outside the range of [Q1–1.5 × IQR, Q3 + 1.5 × IQR] were defined as outliers and excluded. This process resulted in a final dataset of 629 samples. The processed data were then partitioned into a training set (70%) and a test set (30%) using simple random sampling, which were used for model development and parameter tuning, and for evaluating generalization capability, respectively.

#### 2.3.2 Model construction and evaluation

The total sample (*n* = 629) was randomly partitioned into a training set (*n* = 440) and a test set (*n* = 189) at a ratio of 7:3. Given that all participants were male, the gender variable exhibited zero variance and was consequently excluded; the model was thus constructed using the remaining 18 phenotypic features derived from the questionnaire and preprocessing. To assess multicollinearity among predictor variables and prevent their interdependence from compromising model performance, the Variance Inflation Factor (VIF) for these 18 features was calculated prior to modeling using the *variance_inflation_factor* function in the *statsmodels* package. Simultaneously, to ensure balanced learning across categories and enhance the identification of critical minority classes—thereby preventing the model from biasing toward the majority class due to distribution imbalance—the Synthetic Minority Over-sampling Technique (SMOTE) was applied to the training set using the Python library *imblearn*. This process generated synthetic minority samples via interpolation to balance the class distribution, while the test set was maintained in its original imbalanced state to authentically evaluate model performance. Subsequently, seven machine learning models were developed employing diverse algorithmic strategies, including Logistic Regression, XGBoost, LightGBM, Decision Tree, Gradient Boosting, GaussianNB, and K-Nearest Neighbors. To evaluate and compare model performance, 10-fold cross-validation was utilized, and the optimal model was selected based on post-optimization metrics including Accuracy, Precision, F1-score, and AUC. Grid search was further conducted on the optimal model for hyperparameter tuning. Finally, performance on the test set was evaluated using a One-vs-Rest multi-class strategy, integrating metrics such as the Area Under the Receiver Operating Characteristic Curve (AUC), F1-score, Macro-average, Micro-average, and confusion matrices.

#### 2.3.3 Model interpretation

**Figure 1:**
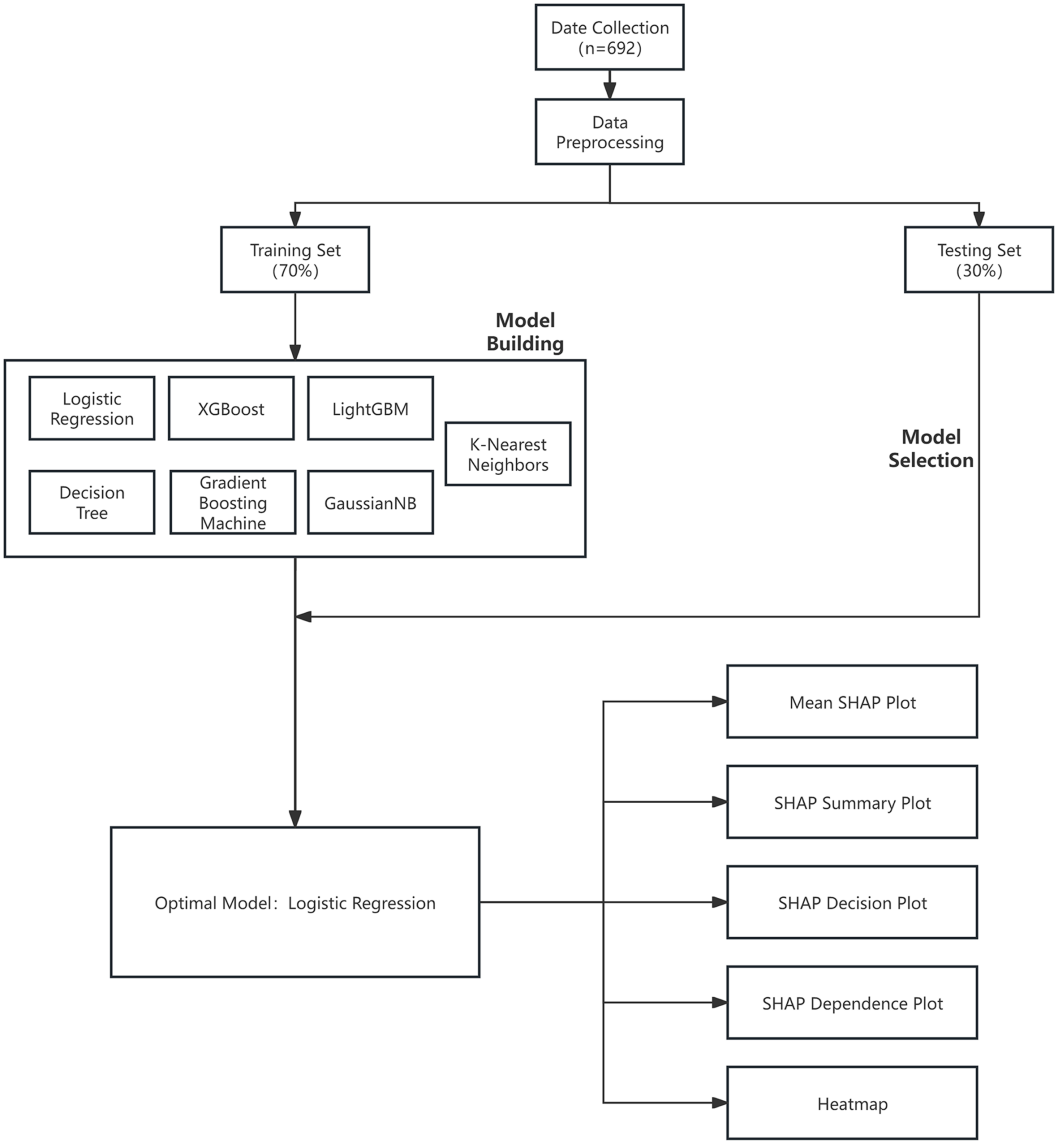
The overall framework of the study in a flowchart.

The interpretability of machine learning remains a significant challenge. To comprehensively elucidate the specific impact and contribution of each feature variable to the final model, we adopted the SHAP method to interpret the optimal black-box model (21). Leveraging principles from game theory, SHAP values treat each feature as a “player” to estimate its specific influence on prediction outcomes. This approach enables a fair allocation of the model’s predictive performance to individual features, thereby explaining the contribution of each feature to individual data points. Specifically, five types of SHAP plots were generated to provide a holistic understanding of feature effects: (1) the Mean SHAP plot (bar chart), to display global feature importance; (2) the SHAP Beeswarm plot, to illustrate the distribution of SHAP values across samples; (3) the SHAP Decision plot, to demonstrate how the model arrives at prediction decisions based on feature values; (4) the SHAP Dependence plot, to show the local impact trends of variations in a single feature’s value on model output within specific craving categories; and (5) the SHAP Heatmap, to visualize overall distribution patterns among features and their impact on model predictions. Figure 1 illustrates the complete workflow of this study.

## 3 Results

### 3.1 Data preprocessing results

This study initially included 692 samples; following data preprocessing, a total of 629 valid samples remained (details provided in Table 1). Specifically, the training set consisted of 282 cases of Low Craving, 135 cases of Moderate Craving, and 23 cases of High Craving, whereas the test set comprised 124, 60, and 5 cases, respectively. No statistically significant differences were observed between the training and test sets regarding demographic characteristics and drug-related clinical features (*p* > 0.05); however, the frequency of drug use differed significantly among different craving categories (*p* < 0.001). The results indicated a substantial class imbalance within the dataset, which could potentially bias the model towards the majority class during training. Consequently, targeted measures were implemented throughout the subsequent model construction and evaluation phases to mitigate this impact. Specifically, during the model construction phase, the SMOTE strategy was employed to address class imbalance. During the evaluation phase, Macro-average AUC and F1-score were utilized to equally weigh the model’s comprehensive discriminative capability across all categories, complemented by the Micro-average AUC metric to assess classification performance at the overall sample level. Furthermore, we focused on analyzing the individual AUC values and confusion matrices for each category (particularly the high-craving group) to specifically evaluate the model’s identification efficacy for critical minority classes. This comprehensive evaluation strategy ensured a holistic and unbiased assessment of the model’s performance on imbalanced data.

**Table 1:** Distribution characteristics of drug users across the training and testing sets.

Category		Training set (*n* = 440)				Testing set (*n* = 189)			
		0 (*n* = 282)	1 (*n* = 135)	2 (*n* = 23)	*p*	0 (*n* = 124)	1 (*n* = 60)	2 (*n* = 5)	*p*
Age [median (IQR)]		42.00 [36.00, 48.00]	41.00 [37.00, 48.00]	44.00 [38.00, 48.50]	0.677	41.00 [36.00, 46.00]	43.00 [35.00, 49.00]	43.00 [39.00, 46.00]	0.482
Education (%)	1	15 (5.3)	5 (3.7)	0 (0.0)	0.134	8 (6.5)	6 (10.0)	0 (0.0)	0.576
	2	43 (15.2)	21 (15.6)	3 (13.0)		16 (12.9)	11 (18.3)	0 (0.0)	
	3	148 (52.5)	86 (63.7)	10 (43.5)		65 (52.4)	28 (46.7)	4 (80.0)	
	4	59 (20.9)	20 (14.8)	9 (39.1)		29 (23.4)	11 (18.3)	1 (20.0)	
	5	13 (4.6)	1 (0.7)	1 (4.3)		5 (4.0)	1 (1.7)	0 (0.0)	
	6	4 (1.4)	2 (1.5)	0 (0.0)		1 (0.8)	3 (5.0)	0 (0.0)	
Marriage (%)	1	90 (31.9)	43 (31.9)	8 (34.8)	0.918	36 (29.0)	19 (31.7)	0 (0.0)	0.285
	2	10 (3.5)	8 (5.9)	1 (4.3)		6 (4.8)	7 (11.7)	0 (0.0)	
	3	104 (36.9)	51 (37.8)	7 (30.4)		40 (32.3)	20 (33.3)	3 (60.0)	
	4	78 (27.7)	33 (24.4)	7 (30.4)		42 (33.9)	14 (23.3)	2 (40.0)	
Occupation (%)	1	14 (5.0)	13 (9.6)	3 (13.0)	0.519	9 (7.3)	2 (3.3)	0 (0.0)	0.61
	2	6 (2.1)	4 (3.0)	0 (0.0)		3 (2.4)	4 (6.7)	0 (0.0)	
	3	45 (16.0)	20 (14.8)	4 (17.4)		18 (14.5)	10 (16.7)	0 (0.0)	
	4	55 (19.5)	22 (16.3)	3 (13.0)		19 (15.3)	6 (10.0)	2 (40.0)	
	5	31 (11.0)	9 (6.7)	2 (8.7)		14 (11.3)	5 (8.3)	0 (0.0)	
	6	26 (9.2)	11 (8.1)	0 (0.0)		12 (9.7)	3 (5.0)	0 (0.0)	
	7	92 (32.6)	51 (37.8)	11 (47.8)		43 (34.7)	27 (45.0)	3 (60.0)	
	8	13 (4.6)	5 (3.7)	0 (0.0)		6 (4.8)	3 (5.0)	0 (0.0)	
Income [median (IQR)]		3000.00 [2000.00, 5000.00]	4000.00 [2000.00, 6000.00]	3000.00 [2000.00, 5000.00]	0.497	3000.00 [2000.00, 6000.00]	3000.00 [2000.00, 6000.00]	5000.00 [4000.00, 5000.00]	0.595
Family (%)	1	166 (58.9)	74 (54.8)	11 (47.8)	0.506	74 (59.7)	28 (46.7)	3 (60.0)	0.411
	2	101 (35.8)	50 (37.0)	9 (39.1)		37 (29.8)	26 (43.3)	2 (40.0)	
	3	15 (5.3)	10 (7.4)	3 (13.0)		13 (10.5)	6 (10.0)	0 (0.0)	
	4	0 (0.0)	1 (0.7)	0 (0.0)					
Duration [median (IQR)]		10.00 [5.00, 15.00]	10.00 [6.00, 15.00]	15.00 [8.00, 20.00]	0.032	10.00 [5.00, 15.50]	10.00 [8.00, 20.00]	20.00 [15.00, 20.00]	0.008
Quantity [mean (SD)]		0.49 (0.44)	0.53 (0.40)	0.91 (1.02)	<0.001	0.60 (0.96)	0.73 (0.61)	0.80 (0.27)	0.556
Time served [median (IQR)]		613.50 [523.25, 644.00]	614.00 [545.50, 643.00]	613.00 [576.50, 670.00]	0.568	612.50 [544.75, 632.25]	613.00 [516.00, 636.00]	731.00 [730.00, 972.00]	0.014
Frequency (%)	1	96 (34.0)	27 (20.0)	2 (8.7)	<0.001	46 (37.1)	11 (18.3)	0 (0.0)	<0.001
	2	102 (36.2)	29 (21.5)	0 (0.0)		38 (30.6)	16 (26.7)	1 (20.0)	
	3	55 (19.5)	40 (29.6)	7 (30.4)		25 (20.2)	14 (23.3)	0 (0.0)	
	4	22 (7.8)	25 (18.5)	7 (30.4)		13 (10.5)	13 (21.7)	1 (20.0)	
	5	7 (2.5)	10 (7.4)	3 (13.0)		2 (1.6)	4 (6.7)	1 (20.0)	
	6	0 (0.0)	4 (3.0)	4 (17.4)		0 (0.0)	2 (3.3)	2 (40.0)	
Methamphetamine [mean (SD)]		0.51 (0.50)	0.53 (0.50)	0.57 (0.51)	0.861	0.48 (0.50)	0.45 (0.50)	0.20 (0.45)	0.444
MDMA [mean (SD)]		0.28 (0.45)	0.28 (0.45)	0.43 (0.51)	0.27	0.24 (0.43)	0.27 (0.45)	0.00 (0.00)	0.413
Amphetamines [mean (SD)]		0.02 (0.13)	0.03 (0.17)	0.09 (0.29)	0.114	0.02 (0.13)	0.02 (0.13)	0.00 (0.00)	0.96
Ketamine [mean (SD)]		0.03 (0.17)	0.03 (0.17)	0.13 (0.34)	0.033	0.03 (0.18)	0.03 (0.18)	0.00 (0.00)	0.92
Heroin [mean (SD)]		0.45 (0.50)	0.47 (0.50)	0.52 (0.51)	0.813	0.48 (0.50)	0.53 (0.50)	0.80 (0.45)	0.312
Snorting [mean (SD)]		0.76 (0.43)	0.73 (0.44)	0.61 (0.50)	0.248	0.76 (0.43)	0.57 (0.50)	0.40 (0.55)	0.011
Injection [mean (SD)]		0.22 (0.41)	0.23 (0.42)	0.39 (0.50)	0.173	0.24 (0.43)	0.37 (0.49)	0.60 (0.55)	0.067
Oral ingestion [mean (SD)]		0.07 (0.25)	0.03 (0.17)	0.13 (0.34)	0.103	0.07 (0.26)	0.13 (0.34)	0.00 (0.00)	0.315

### 3.2 Model construction results

The results of the feature multicollinearity analysis indicated that there was no significant multicollinearity among the 18 predictor variables included in this study. As shown in Table 2, the Variance Inflation Factors (VIF) for the variables ranged from 1.01 to 2.82, all of which were well below the empirical threshold (VIF < 5). This suggests that the features possess good independence without serious information redundancy, making them suitable for the subsequent construction of classification models.

**Table 2:** Results of feature collinearity analysis.

Variable	VIF
Frequency	2.2814
Age	1.5540
Quantity	1.0885
Heroin	2.8202
Duration	2.7018
Income	1.0861
Ketamine	1.3166
Methamphetamine	2.0553
Snorting	2.1400
Time_served	1.0129
Family	1.1571
Education	1.1815
Occupation	1.1475
Oral_ingestion	1.1045
Injection	2.5999
Amphetamines	1.3502
MDMA	1.1889
Marriage	1.1432

This study further developed seven machine learning models and conducted 10-fold cross-validation to comprehensively evaluate the robustness of each model following SMOTE resampling across multiple dimensions, including Accuracy, Precision, Recall, F1-score, and AUC. The performance of each model during the 10-fold cross-validation is summarized in Table 3. To present the complete details of the model evaluation, specific performance metrics for the seven models across each fold are provided in Supplementary Table 2. The results indicated that models such as Logistic Regression, Gradient Boosting, and eXtreme Gradient Boosting (XGBoost) exhibited superior overall performance, with minimal differences observed in key metrics. Specifically, Logistic Regression achieved an AUC of 0.9079, ranking highest among all models, while performing on par with the strongest algorithms across other key metrics. This suggests that Logistic Regression possesses classification performance comparable to that of more complex models. Considering its structural simplicity, low computational cost, and superior clinical interpretability—which are more conducive to exploring addiction mechanisms and facilitating clinical translation—Logistic Regression was ultimately identified as the optimal prediction model in this study.

**Table 3:** Comparison of model performance on the training set using 10-fold cross-validation.

Model	Accuracy	Recall (weighted)	Precision (weighted)	F1-score (weighted)	AUC (one-vs-rest)	Matthews correlation coefficient	Negative log loss
Logistic regression	0.8246	0.8246	0.8019	0.8082	0.9079	0.5236	−0.4235
XGBoost	0.8271	0.8271	0.8237	0.8214	0.9067	0.5456	−0.5563
LightGBM	0.8234	0.8234	0.8206	0.8175	0.8968	0.5360	−0.7522
Decision tree	0.8004	0.8004	0.8014	0.7971	0.7864	0.4887	−5.8622
Gradient boosting	0.8476	0.8476	0.8481	0.8417	0.9078	0.6020	−0.4203
GaussianNB	0.7036	0.7036	0.7299	0.7136	0.7762	0.2916	−0.9131
KNN	0.7401	0.7401	0.6350	0.6651	0.5497	0.0657	−4.1111

For the optimal Logistic Regression model, we specifically conducted a grid search targeting the regularization parameter *C* (0.01, 0.1, 1, 10, 100), penalty term (*l1, l2, elasticnet*), solver (*lbfgs, saga*), multi-class strategy (*ovr, multinomial*), and maximum number of iterations (100, 200, 500, 1,000). The final optimal parameter configuration was determined as follows: *C* = 1, *penalty* = ‘l2’, *solver* = ‘lbfgs’, *multi_class* = ‘multinomial’, and *max_iter* = 1,000.

### 3.3 Classification reliability test results

The internal consistency analysis revealed that the Cronbach’s *α* coefficient for the total scale was 0.9796, significantly exceeding the conventional threshold of 0.80. This indicates an exceptionally high level of inter-item reliability (see Supplementary Table 3). Simultaneously, to verify whether the threshold settings for categorizing the total score of the self-developed scale into three classes strictly adhered to the grading rationale of the VAS, we calculated the consistency between the current classification rules and the theoretical logical classification of the VAS. As presented in Table 4, the results yielded a Kappa coefficient of 1.0 (*p* < 0.001). This demonstrates that the classification rules established based on the total score mathematically achieved a severity stratification perfectly corresponding to the VAS grading standards, thereby ensuring that the target variables for the subsequent machine learning models possess a clear and consistent theoretical reference.

**Table 4:** Agreement test results between the drug craving scale classification and the VAS classification.

Method	Value	ASE	*z*	Pr (>|*z*|)
Unweighted	1	0	Inf	0
Weighted	1	0	Inf	0

### 3.4 Model evaluation and comparison

**Figure 2:**
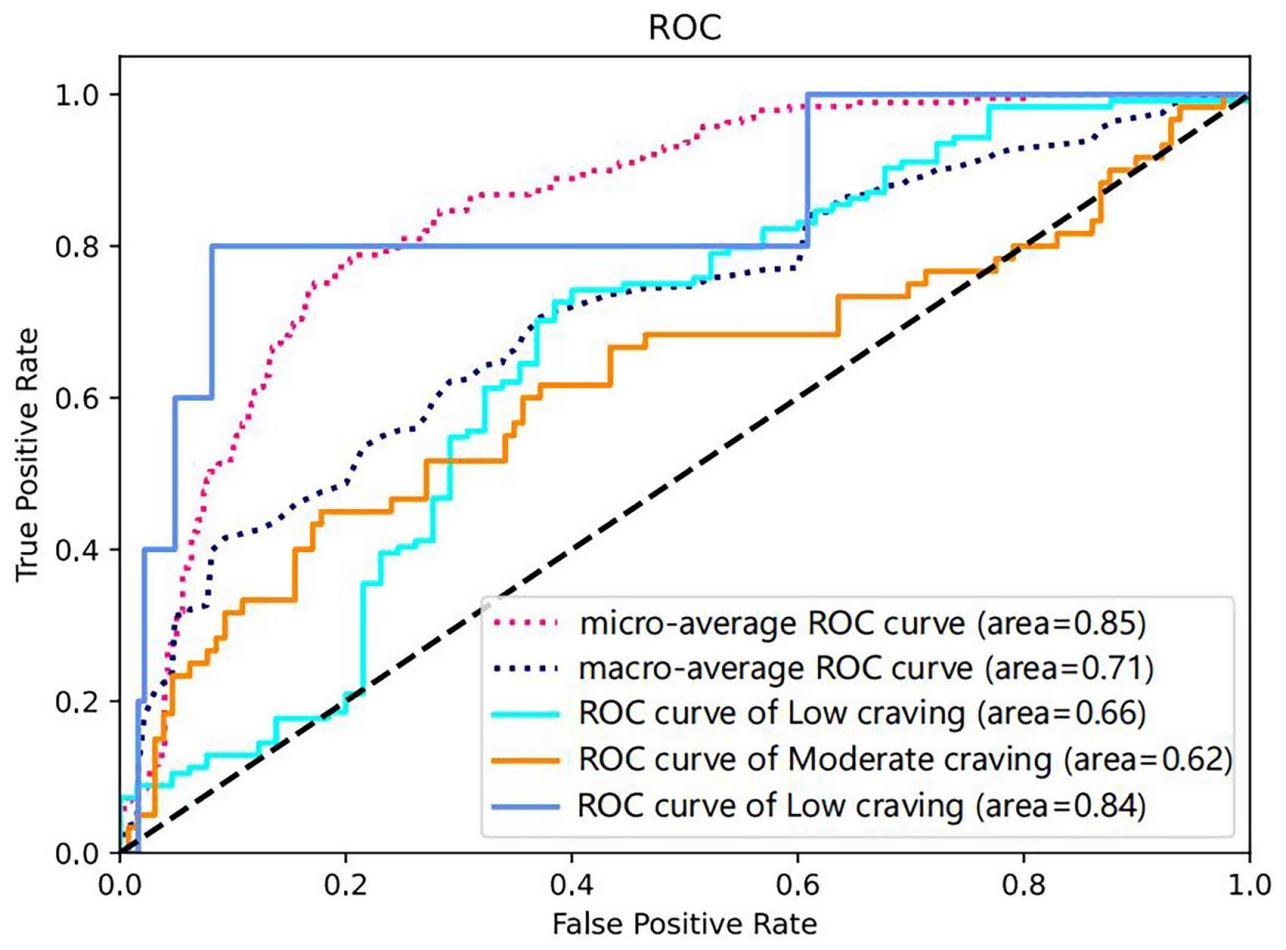
ROC curve of the logistic regression model for drug craving classification on the test set.

**Figure 3:**
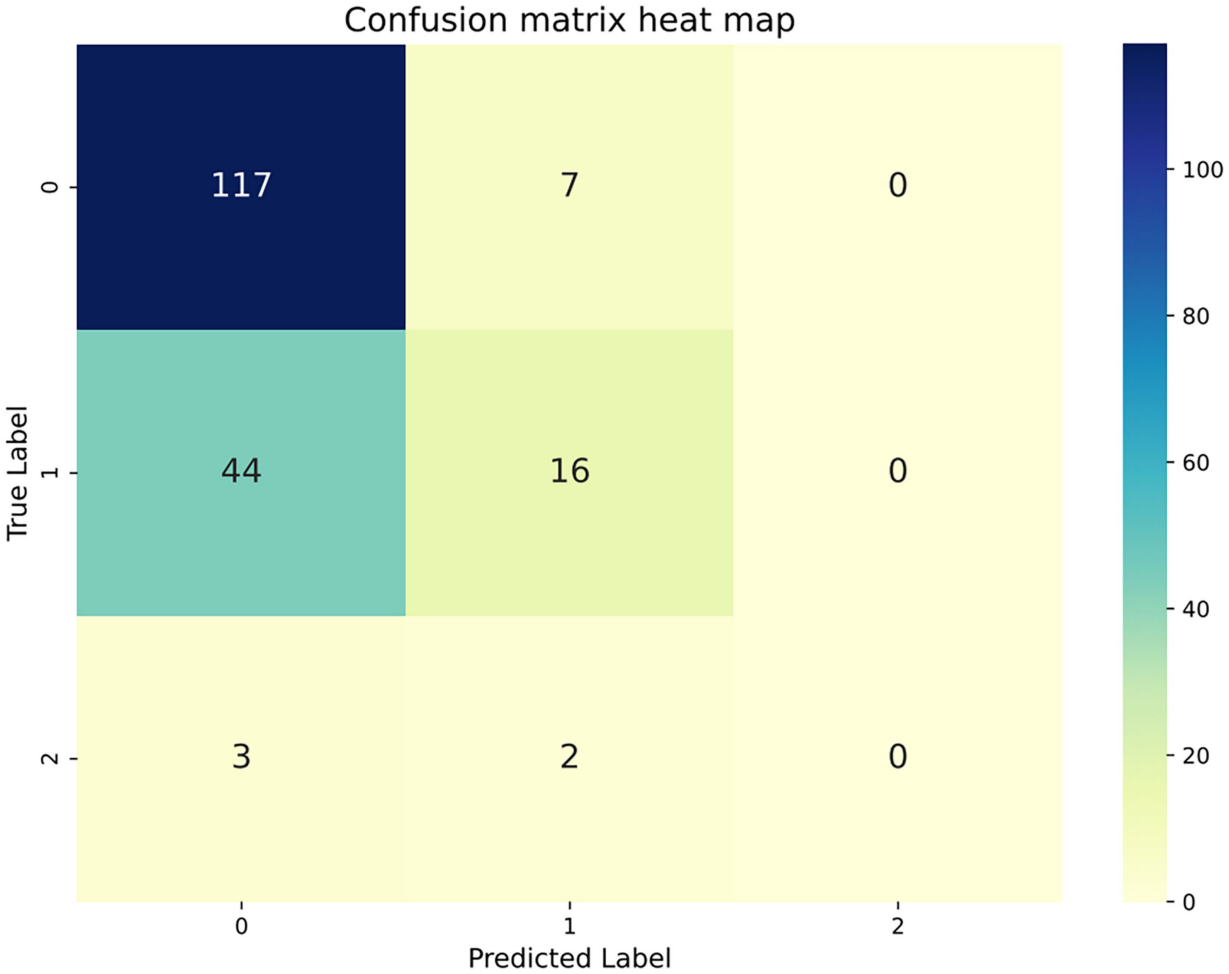
Confusion matrix of the logistic regression model for three-class classification on the test set.

Following the identification of Logistic Regression as the optimal model based on 10-fold cross-validation on the training set, this study further applied it to a completely independent test set to validate its generalization performance under real-world data distribution. The results indicated that on the test set, the Logistic Regression model achieved an accuracy of 66.13%, an F1-score of 0.54, a Macro-AUC of 0.71, and a Micro-AUC of 0.85 (see Table 5; Figure 2; ROC curves for the other six models are presented in Supplementary Figure 1, and confusion matrices in Supplementary Figure 2). Given the class imbalance present in the test set, relying solely on accuracy could be misleading; therefore, model evaluation requires a multi-dimensional analysis. Overall, the model’s Micro-AUC reached 0.85, indicating good overall discriminatory capability across all samples; meanwhile, the Macro-AUC of 0.71 suggests that the model maintains a certain level of recognizability for each category, not completely ignoring other categories due to the predominance of Low Craving samples. It is particularly notable that the model’s identification capability for the three craving categories—High, Moderate, and Low—was not uniform, with AUC values of 0.84, 0.62, and 0.66, respectively. This distribution suggests that the model shows encouraging preliminary sensitivity in identifying the High Craving group, which is of greater importance for clinical intervention. However, analysis combined with the confusion matrix (Figure 3) revealed that although the model successfully identified 3 out of the only 5 High Craving samples in the test set, demonstrating encouraging preliminary sensitivity, the absolute robustness of its classification boundary remains to be further verified with larger samples due to the extremely small scale of this category.

**Table 5:** Performance evaluation of different machine learning models on the testing set.

Model	F1-score	Accuracy
Logistic regression	0.54	0.6613
XGBoost	0.62	0.6036
LightGBM	0.58	0.6351
Decision tree	0.53	0.5343
Gradient boosting	0.62	0.5932
GaussianNB	0.31	0.2222
KNN	0.52	0.6508

### 3.5 SHAP analysis results

**Figure 4:**
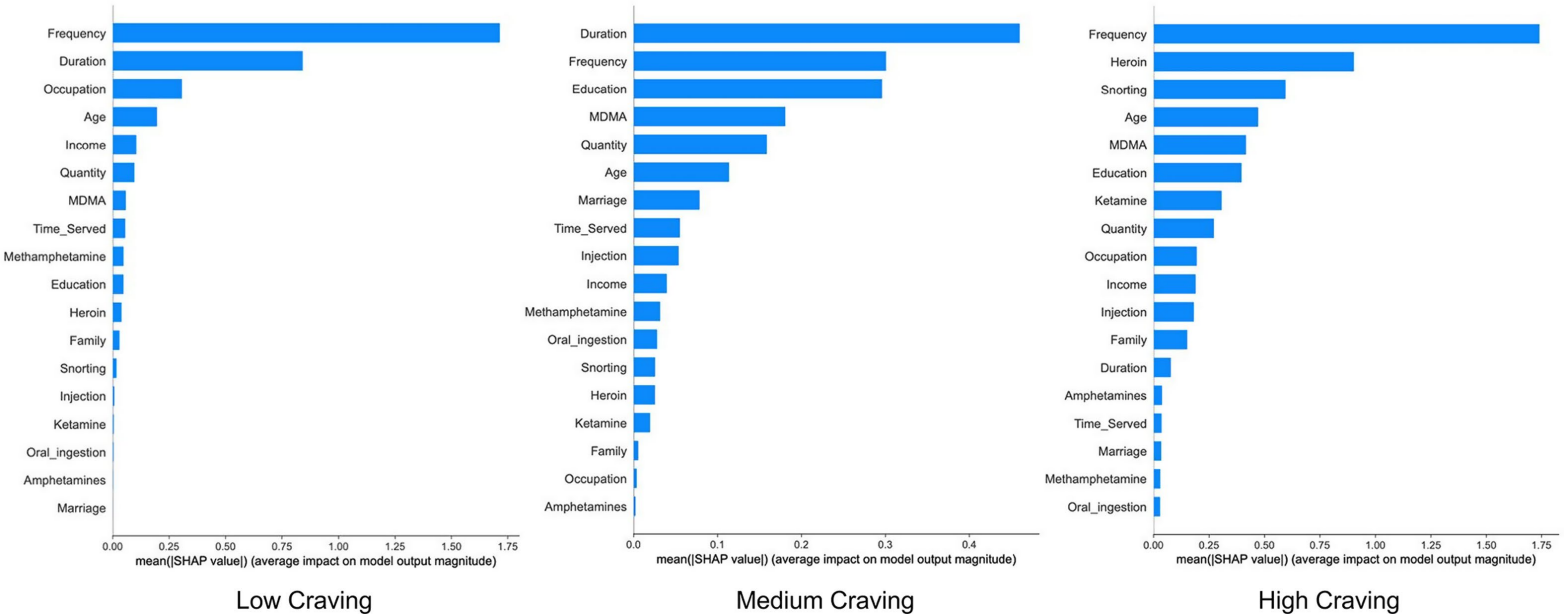
SHAP feature importance bar plot of the logistic regression model.

We utilized the SHAP method to identify the key influencing factors of the Logistic Regression model and ranked the features based on the magnitude of their SHAP value contributions. As illustrated in Figure 4, the results demonstrated a high correlation between Frequency (frequency of drug use), Duration (years of drug use), and Drug Type 5 (Heroin) and the prediction of drug craving levels.

**Figure 5:**
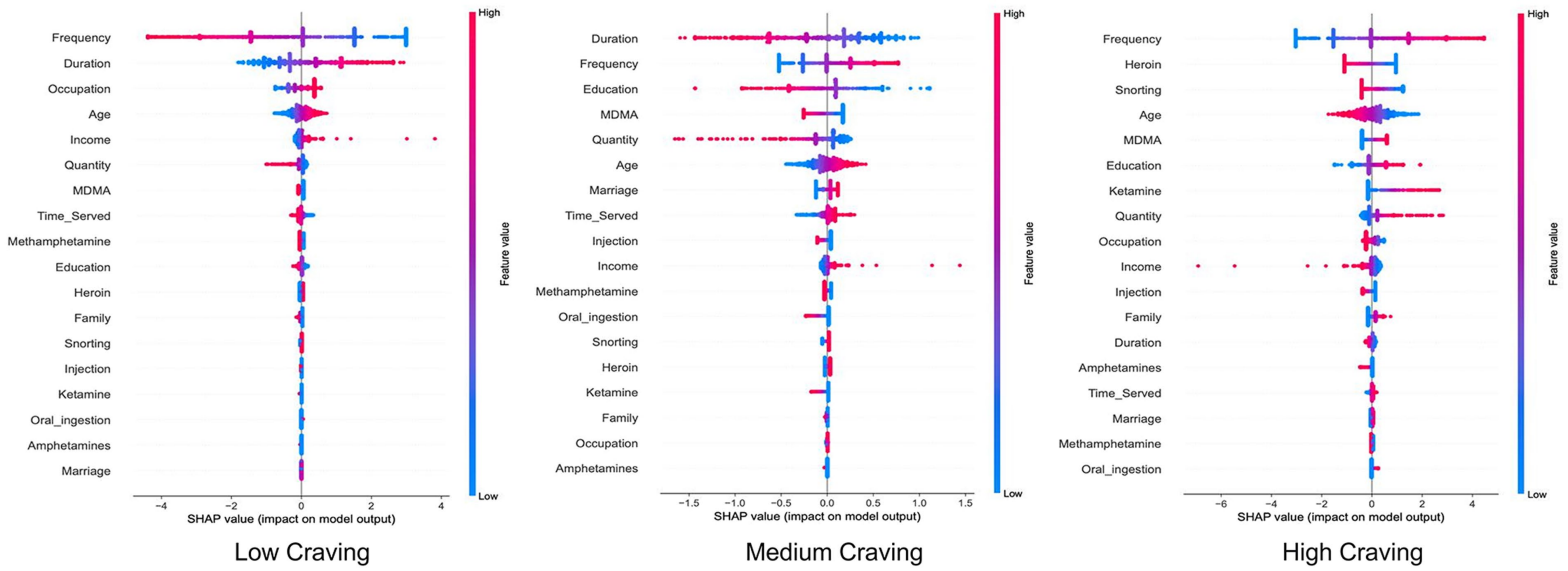
SHAP summary plot of the logistic regression model.

The SHAP Beeswarm plot intuitively visualizes the importance of each variable and its positive or negative impact on the prediction results of the Logistic Regression model. As shown in Figure 5, each row in the plot represents a variable, with the horizontal axis denoting the SHAP value; features are ranked in descending order based on their importance. Each dot represents an individual sample, where red indicates a higher feature value and blue indicates a lower feature value. A positive SHAP value suggests that the feature contributes to increasing the model’s prediction output, whereas a negative SHAP value implies a negative impact. Taking “Frequency” (frequency of drug use)—a feature with significant influence on prediction results—as an example, its SHAP value distribution reveals a positive correlation: a higher frequency of drug use corresponds to a greater likelihood of being classified as Moderate or High Craving. Furthermore, for high-importance features such as “Education Level,” SHAP values are widely distributed across both positive and negative intervals. This indicates that its influence serves as neither a simple risk factor nor a protective factor; rather, its contribution varies with the feature value itself, shifting from a risk factor at lower education levels to a protective factor at higher levels. These findings not only validate the relevance of sociodemographic characteristics but also quantify their specific contribution weights within the model via SHAP values.

**Figure 6:**
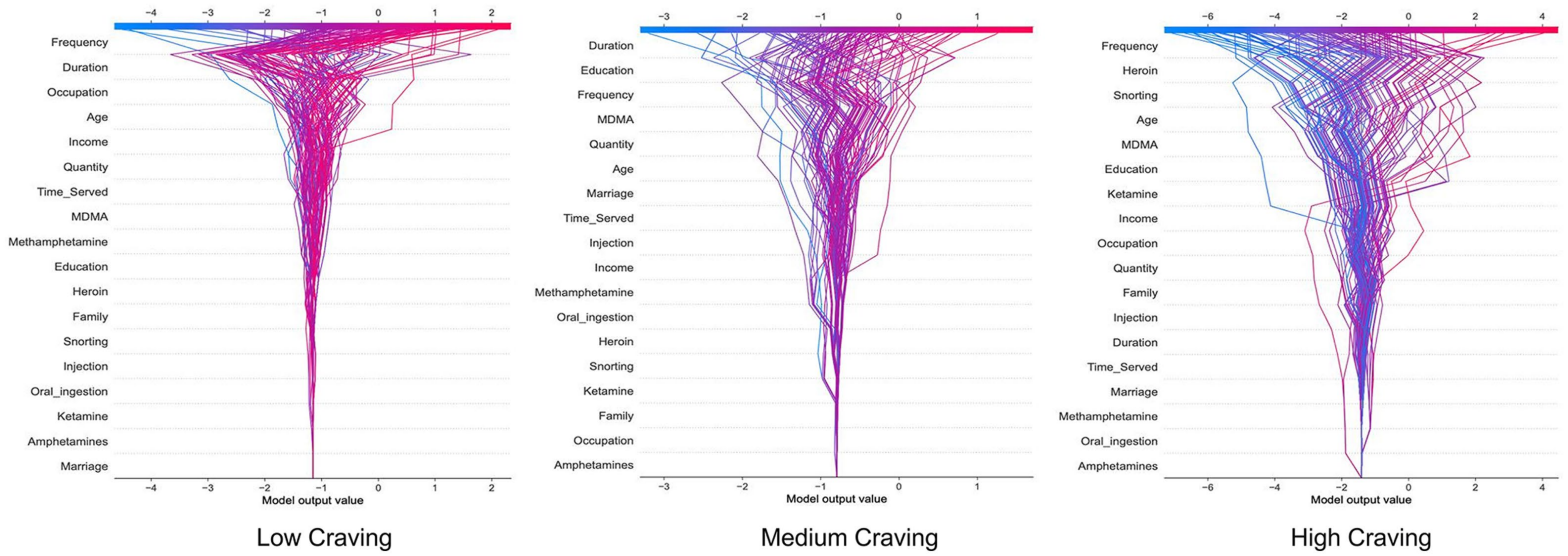
SHAP decision plot of the logistic regression model.

The SHAP decision plot provides a visual representation of the magnitude and direction of each feature’s contribution to the model’s prediction results. As shown in Figure 6, the vertical axis represents different feature variables, ranked in descending order of importance, while the horizontal axis denotes the model output value, with the gray vertical line in the center indicating the model’s baseline prediction value. The colored lines in the plot represent the prediction paths for different samples: each line originates from the baseline value and accumulates the SHAP values of specific features from bottom to top, ultimately reaching the final prediction score at the top. The color of the lines reflects prediction intensity, where red indicates a higher output value (higher likelihood of being predicted as the target class) and blue corresponds to a lower output value (lower classification probability). This plot effectively captures the specific contribution of various feature combinations on prediction outcomes, further validating the significant influence of features such as drug use frequency, duration of use, and heroin use on the prediction of craving levels.

**Figure 7:**
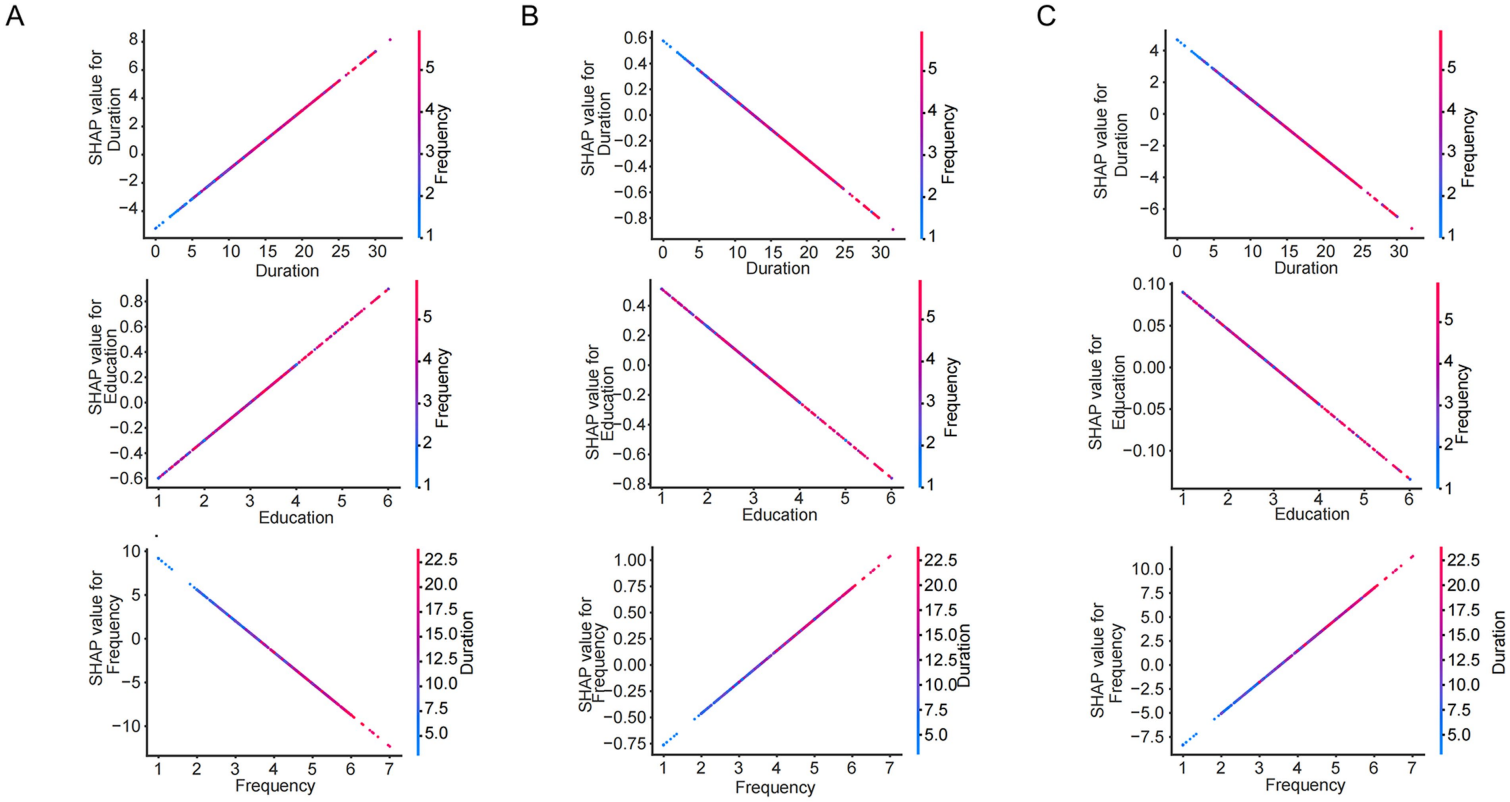
SHAP dependence plots of representative features of the logistic regression model. Panels **(A)**, **(B)**, and **(C)** correspond to Low, Moderate, and High Craving groups, respectively.

To further elucidate the differential impacts of specific features across craving states, we conducted an in-depth analysis using SHAP dependence plots. As illustrated in Figure 7 (showcasing representative features: Frequency, Duration, and Education) and Supplementary Figure 3 (presenting the complete feature set), distinct contribution patterns were observed between the Low and High Craving groups (Figures 7A–C correspond to Low, Moderate, and High Craving groups, respectively). Notably, drug-use behavioral features, specifically Frequency and Duration, exhibited a pronounced positive correlation. In the High Craving group, SHAP values escalated significantly with increasing feature values, identifying high frequency and prolonged usage as critical drivers of intensified craving. Conversely, within the Low Craving group, SHAP values for these features declined precipitously as values increased, suggesting that as usage frequency and duration increase, the probability of maintaining a low craving state diminishes proportionally. Furthermore, sociodemographic characteristics, exemplified by Education Level, primarily manifested as protective factors of varying magnitude. For the Low Craving group, higher socioeconomic status was associated with elevated positive SHAP values, acting as a stabilizer. However, in the High Craving group, while these features retained a negative impact (tending to lower craving scores), their absolute SHAP values were markedly attenuated. This indicates that in the advanced stages of addiction, the protective efficacy of social factors may be overshadowed by the intensity of drug-use behaviors.

**Figure 8:**
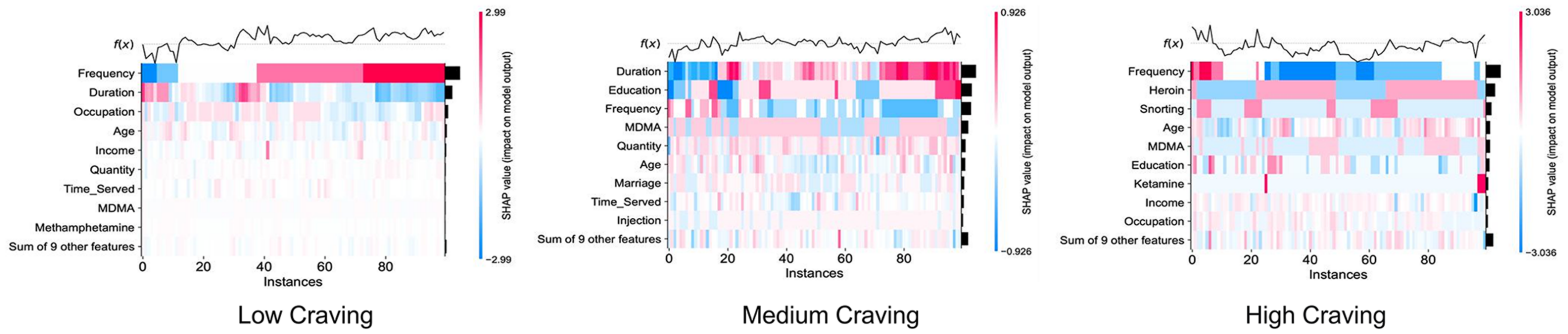
SHAP heatmap of the logistic regression model.

The SHAP heatmap of the Logistic Regression model demonstrates the impact of different features on model predictions from both sample and feature dimensions through intuitive variations in color and position. As shown in Figure 8, the *X*-axis represents the sample sequence, while *f*(*x*) above the *X*-axis denotes the summation of SHAP values for each sample, representing the degree of deviation from the mean. The left side of the *Y*-axis lists the feature names, and the bar chart on the right displays the summation of SHAP values across the feature dimension. The intensity of the color blocks indicates the magnitude of SHAP values: red represents high SHAP values that positively increase classification probability, while blue represents low SHAP values that suppress classification probability. From the sample dimension, the *f*(*x*) curve at the top of the *X*-axis intuitively presents the overall prediction shift of the samples. Taking Frequency in the Low Craving group as an example, the color blocks are intensely red, indicating that these samples are significantly positively influenced by drug use frequency; furthermore, the summation of sample SHAP values *f*(*x*) is above the average line, identifying them as samples with high predictive confidence. From the feature dimension, the bar chart further confirms a high correlation between Frequency, Duration, Heroin, and the prediction of drug craving levels.

## 4 Discussion

By integrating machine learning with the SHAP interpretability framework, this study systematically constructed a classification prediction model for drug craving to assess the potential for post-detoxification relapse among drug users, quantitatively analyzed the multi-dimensional influencing factors of drug craving, and elucidated the specific contribution weights of each feature to prediction outcomes. The results demonstrated that the Logistic Regression model yielded the optimal performance among the seven algorithms (test set accuracy: 0.66; F1-score: 0.54). In particular, it exhibited the highest specificity in the Low Craving category (AUC = 0.66), and its discrimination metrics (Macro-AUC: 0.71; Micro-AUC: 0.85) significantly outperformed those of the comparative algorithms. This indicates that, within the context of this study, Logistic Regression achieved the best balance between fitting capability and generalization stability. SHAP analysis further revealed that Frequency, Duration, and Heroin use are the core predictors of craving levels. Drug craving is collectively influenced by multiple factors; our findings show that drug-use behavioral features (e.g., frequency, duration, type) and sociodemographic factors exert significant yet differentiated contributions to model prediction. This discovery addresses the limitations of traditional analyses that focus solely on coefficient significance; by visualizing feature contributions through SHAP values, it provides an intuitive, quantitative perspective for understanding the factors associated with craving.

Logistic Regression exhibited the optimal comprehensive performance among the seven machine learning models constructed in this study, achieving a test set accuracy of 66.13%. Although this figure leaves room for improvement in absolute terms, it must be objectively evaluated within the research context: first, drug craving, as a complex psychological construct, possesses a dynamic nature that is inherently difficult to fully capture relying solely on phenotypic features; second, the significant class imbalance in the dataset (with only 28 cases in the High Craving group) constrained further improvements in overall discriminative capability; and third, the three-class prediction task itself entails higher complexity compared to binary classification. Nevertheless, Logistic Regression demonstrated distinct advantages in critical dimensions: it displayed encouraging sensitivity in identifying high-craving individuals at high risk of relapse (AUC = 0.84). However, given the very small number of high-craving samples in the test set, the robustness of the model’s performance on this minority class remains exploratory and requires confirmation in larger datasets. Its comprehensive performance was the most robust in the cross-sectional comparison of the seven algorithms. More importantly, the core objective of this study was not merely to pursue prediction precision, but to construct a classification framework that balances predictive efficacy with interpretability. The central innovation of this study lies in the methodological integration—combining machine learning prediction with SHAP interpretability analysis to provide intuitive visualization of feature contributions. Within this framework, the Logistic Regression model serves as an ideal vehicle due to its superior interpretability. Through SHAP analysis, we successfully translated the model’s prediction results into quantitative interpretations of key drivers (e.g., drug use frequency, duration), clearly demonstrating the marginal contributions and positive or negative impacts of these factors on prediction probabilities as their values change during the decision-making process. This deepening from “prediction” to “interpretation” transforms the model from a black box into a transparent tool that, upon further validation, could be capable of providing clinicians with clear intervention targets and decision-making support, which holds greater practical significance for drug rehabilitation practice than the mere pursuit of accuracy improvement.

Furthermore, the results of this study align deeply with classic theoretical frameworks of addiction neurobiology, and through SHAP analysis, provide pivotal quantitative support consistent with these theoretical perspectives from the perspective of predictive modeling. SHAP values explicitly identified drug use frequency and duration as the most core predictors, which aligns with the hypothesis of neuroadaptive changes induced by long-term drug exposure, such as dopamine D2 receptor downregulation, inhibition of prefrontal cortex function, and dysregulation of glutamatergic signaling in the nucleus accumbens (22, 23). The high weights assigned to duration and frequency by SHAP analysis may reflect the progressive impact of cumulative exposure on addiction severity, quantitatively capturing the trend of gradual escalation of craving severity with cumulative exposure. Notably, this study overcomes the limitations of previous research; while Everitt et al. proposed the “habit-to-compulsion” transition theory based on animal experiments (24), such paradigms rooted in operant conditioning—though establishing the neural basis of habituation in addictive behaviors—struggle to quantify the dynamic contributions of human-specific, multi-dimensional social behavioral factors. In contrast, this study successfully quantified the specific contributions of features like drug type and duration via machine learning. Our computational framework thus constructs a bridge upon this neural foundation toward understanding complex human addictive behaviors. Additionally, previous studies suggest that low-frequency users retain partial prefrontal cortex regulatory capabilities (e.g., executive control and impulse inhibition), rendering their craving levels more susceptible to modulation by environmental cues (25). This mechanism provides a theoretical explanation for the phenomenon observed in our SHAP decision plots, where social factors contribute to adjusting the classification probability. Accordingly, this study further conceptualizes drug-use behaviors and sociodemographic characteristics as stable risk drivers and context-dependent moderators, respectively, providing a direct basis for subsequent stratified precision interventions.

In terms of clinical translation, this study demonstrates potential application value by proposing a framework to translate model predictors into stratified intervention strategies. The Logistic Regression model maintained an accuracy of 66.13% even under conditions of class imbalance; this robustness underscores its potential as a preliminary screening tool, though its broad clinical applicability requires further external validation across more diverse, multi-center populations. Specifically, based on the indisputable core status of “Frequency” in the model, we recommend using high-frequency drug use as a key indicator for risk stratification, providing a focal point for intervention strategies: individuals with high-frequency and long-term drug use may require Medication-Assisted Treatment (MAT) (e.g., Methadone) combined with Cognitive Behavioral Therapy, thereby achieving dual regulation of reward pathways (26). Secondly, the significant contribution of social characteristics suggests the complexity of intervention pathways. The inhibitory effects of occupational stability and education level on craving escalation are consistent with the view proposed by Moos (27) that these factors may buffer the risk of craving escalation by enhancing social resources. Most innovatively, the SHAP decision plot breaks the limitations of traditional single-dimensional assessment, intuitively displaying the risk profiles of complex individuals, such as those with high education but high drug use frequency. For such contradictory feature combinations, the model can quantify the offsetting or cumulative effects of various factors, thereby guiding clinicians to formulate personalized plans covering occupational stress management and neurocognitive training (28), rather than applying a one-size-fits-all intervention template. This provides solid empirical support from an algorithmic level for constructing a “Bio-Psycho-Social” integrated detoxification framework.

Methodologically, this study systematically analyzed the factors influencing drug craving by integrating multiple machine learning algorithms with the SHAP framework. This “dual-track” approach of “algorithm optimization coupled with interpretability enhancement” not only improved the model’s predictive performance but also deepened the understanding of addiction mechanisms. Compared to traditional linear models, machine learning not only captured the differential effects of drug use frequency but also quantified the joint contributions of social factors and bio-behavioral features to the model output. Furthermore, addressing the common issue of class imbalance in clinical data, we incorporated the SMOTE oversampling technique during the training phase; this effectively enhanced the sensitivity and identification robustness for the minority class (High Craving group) while preserving the real distribution of the test set, thereby overcoming the limitations of single resampling or relying solely on metric correction. This breakthrough aligns highly with the developing trend of “Explainable AI (XAI)” in medicine (29); as noted by Hassabis et al. (30), SHAP technology, by enhancing model transparency, can effectively foster clinician trust in and application of complex algorithms. Additionally, the adoption of a 10-fold cross-validation strategy combined with grid search significantly optimized model hyperparameters, resolving the issue of poor generalization capability caused by parameter selection bias in traditional research (14), thereby boosting the accuracy on the test set to 66.1%. Moreover, the SHAP global analysis focused on the specific contributions of drug use frequency, duration, drug type, and various social factors on craving, bridging the gap left by previous studies that prioritized biological mechanisms (12).

However, this study is subject to certain limitations. First, regarding sample representativeness, all participants were male abstainers from Hunan Province; this exclusion of females overlooks potential influences of hormonal cycles on craving patterns—for instance, estrogen is known to enhance impulse control by modulating prefrontal-limbic connectivity (31). Furthermore, the latest World Drug Report by the United Nations Office on Drugs and Crime (UNODC) explicitly highlights significant regional disparities in drug abuse patterns globally (2); consequently, this gender homogeneity and regional confinement may introduce bias into the conclusions and constrain the model’s generalization capability. Second, although the application of the SMOTE technique during model construction effectively mitigated learning bias caused by class imbalance, two limitations remain due to the inherent characteristics of the data distribution: first, the scarcity of raw samples in the High Craving category (*n* = 28) restricts the model’s generalization boundaries under extreme feature combinations and may mask certain subtle interactions; second, the use of simple random sampling rather than stratified sampling during the data partitioning phase, while not undermining the model’s core efficacy, may still induce slight instability in the test set distribution. Additionally, the current classification criteria are fundamentally exploratory pending external validation. While they align with VAS logic and demonstrate high internal consistency, they have not yet been externally calibrated against participants’ subsequent relapse behaviors or clinical diagnoses, highlighting the need to validate this internally defined threshold system externally. Finally, the current model relies solely on static questionnaire data, failing to integrate three critical categories of biological information: longitudinal neuroimaging data (e.g., fMRI) which can reveal dynamic associations between anterior cingulate cortex (ACC) activity and craving intensity (32); epigenetic markers (e.g., DNA methylation) which can reflect the biological cumulative effects of long-term drug exposure (33); and multi-modal data combining fMRI with behavioral metrics which can significantly enhance the neurobiological explanatory power of craving predictions (34). The absence of these data dimensions makes it difficult for the model to fully capture the multi-scale biological interplay underlying addictive behaviors.

In response to the aforementioned limitations, future work can expand in the following directions: First, regarding sample representativeness, future studies should incorporate gender-stratified analyses and simultaneously collaborate with drug rehabilitation institutions in other provinces and cities to conduct multi-center validation, thereby enhancing model generalizability. Second, addressing the significant class imbalance, future research should prioritize the targeted recruitment of real clinical samples from the High Craving group to resolve distribution discrepancies at the source; furthermore, stratified sampling strategies should be prioritized during data partitioning to ensure consistent distribution proportions across training and test sets, thereby improving evaluation robustness. Meanwhile, optimization strategies at the algorithmic level, such as Cost-Sensitive Learning, should be explored to mitigate potential noise introduced by synthetic samples (35). Third, to bridge the current gap in biological interpretation, multi-modal data (e.g., fMRI, epigenetic markers) can be integrated to bolster the model’s neurobiological explanatory power. Finally, wearable device-based real-time monitoring tools (e.g., Heart Rate Variability, Galvanic Skin Response) (36) combined with Ecological Momentary Assessment (EMA) can be developed to track temporal fluctuation patterns of craving (37), providing early warning and precision intervention support for community-based rehabilitation.

## 5 Conclusion

This study successfully constructed and validated a machine learning-based classification prediction model for drug craving. The Logistic Regression model, demonstrating optimal predictive performance and interpretability in handling class-imbalanced data, was identified as the optimal model. Through SHAP interpretability analysis, we systematically quantified, for the first time, the importance ranking of core features such as “Frequency,” “Duration,” and “Heroin,” along with their specific contributions to the risk probabilities of distinct craving categories. This model not only serves as a preliminary quantitative tool for screening individuals at high risk of relapse, but more importantly, offers a quantitative perspective for understanding the complex influencing factors of drug craving. Following rigorous external validation across independent and diverse populations, it may provide a crucial scientific basis and data support for formulating personalized, precision intervention strategies for drug rehabilitation in the future.

## Data Availability

To ensure participant privacy and comply with ethical requirements, the anonymized data supporting the findings of this study are available from the corresponding author upon reasonable request.
