# Supplementary figures and images for: A machine learning approach with SHAP interpretability for classifying drug craving levels
Source: Front Public Health. 2026 May 11;14:1752380. doi: 10.3389/fpubh.2026.1752380 (PMC13199168; doi:10.3389/fpubh.2026.1752380)

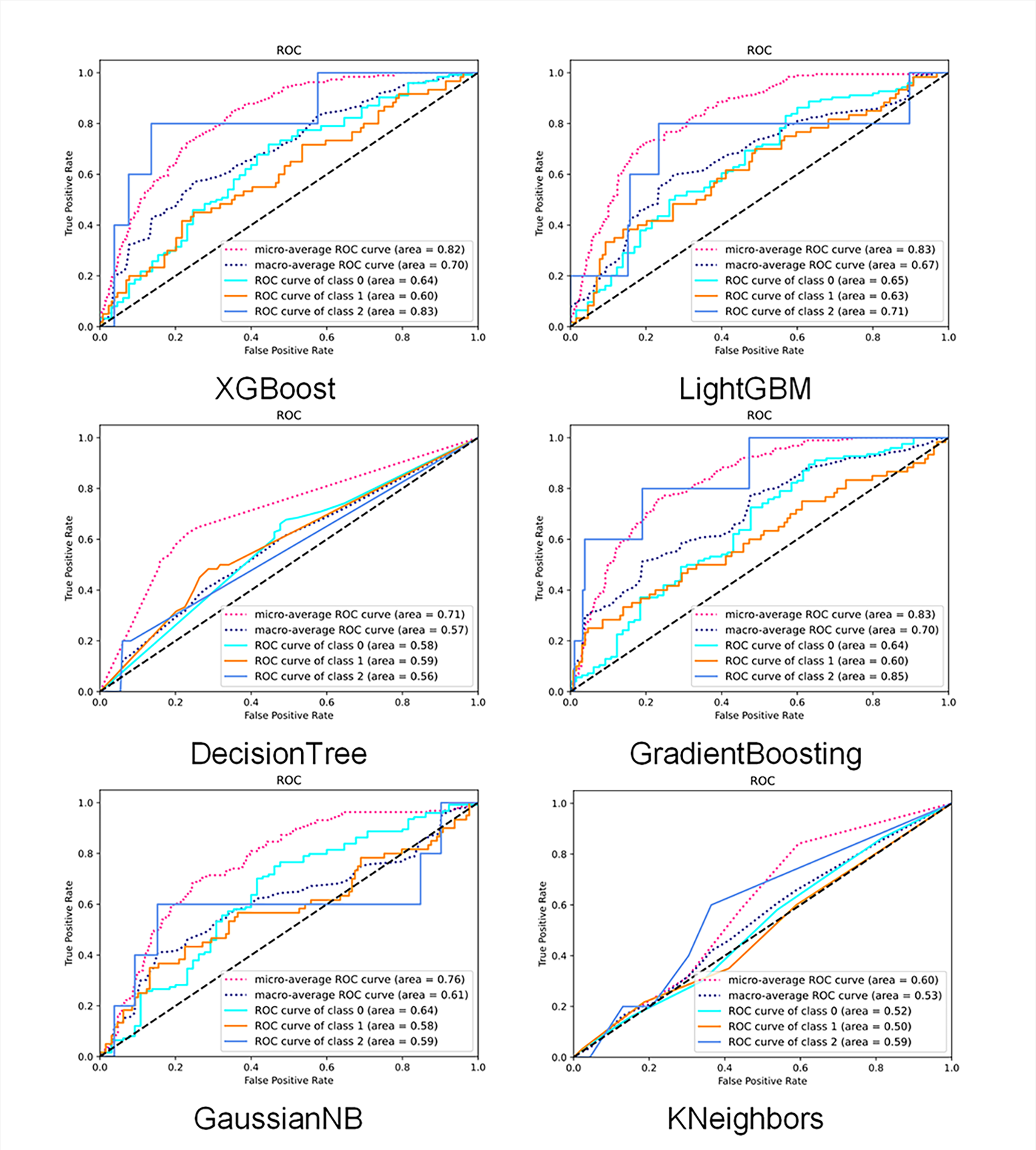

Supplement: Supplementary file 1 [file Image_1.tif]

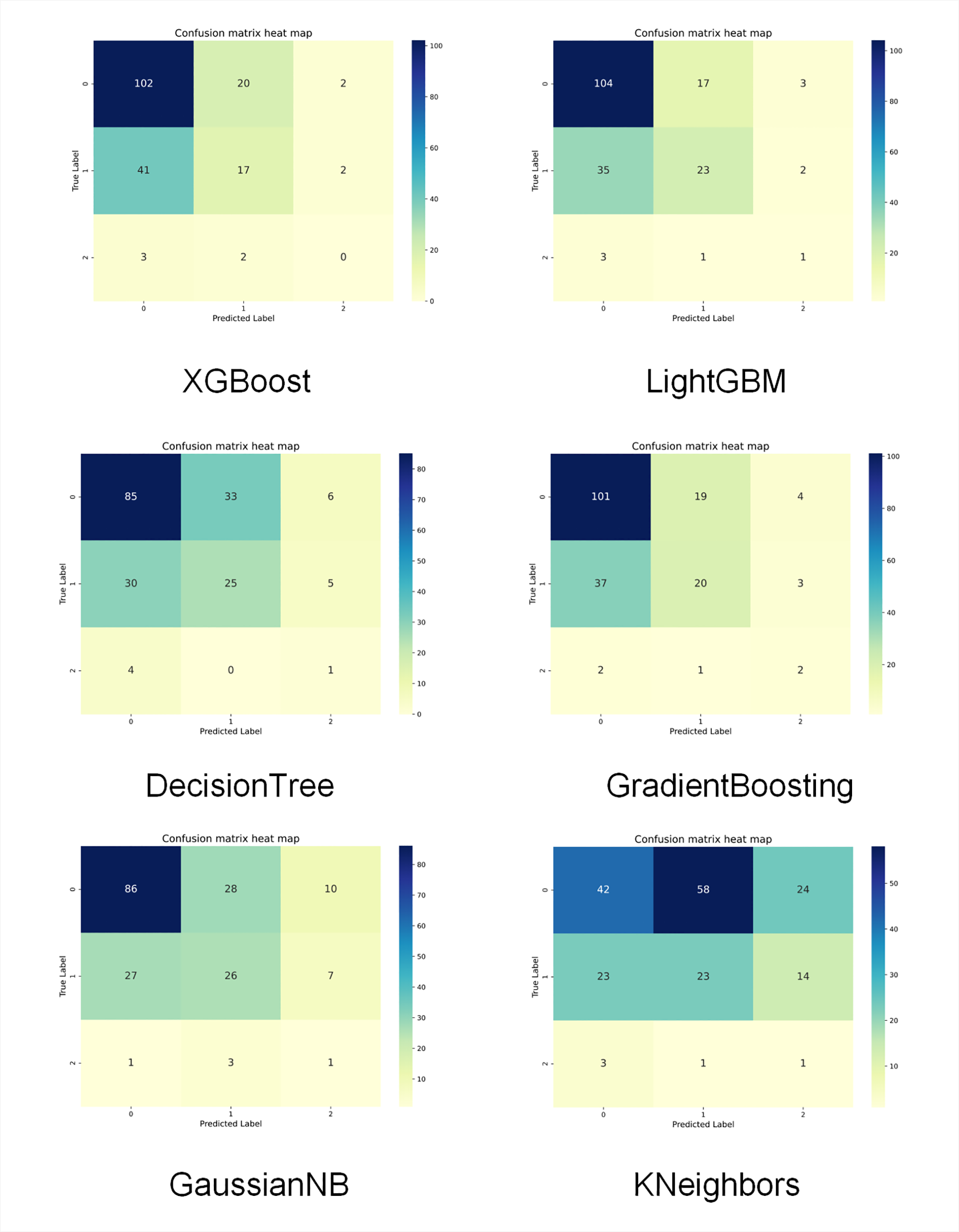

Supplement: Supplementary file 2 [file Image_2.tif]

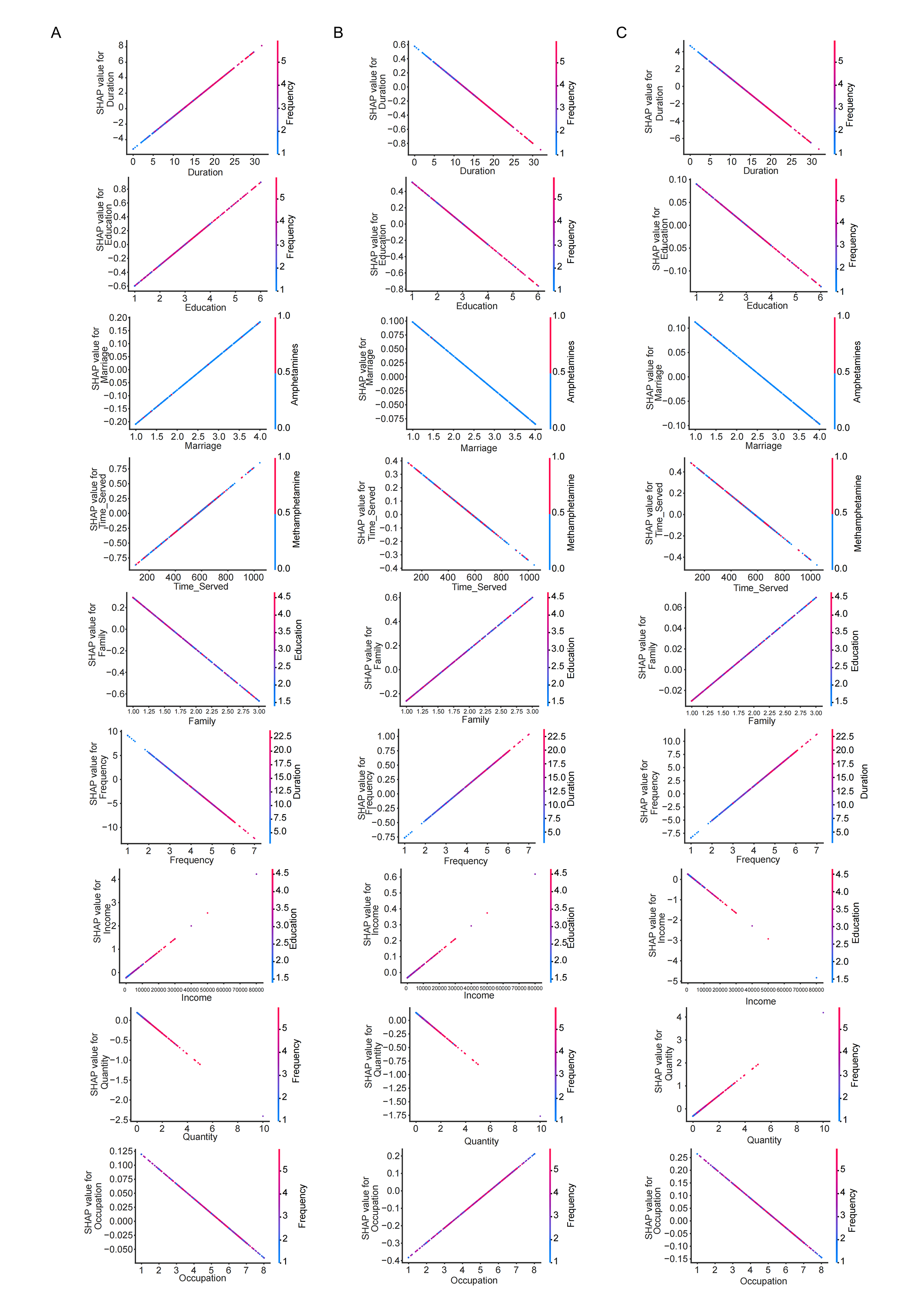

Supplement: Supplementary file 3 [file Image_3.tif]
