# Supplementary material for: A machine learning approach with SHAP interpretability for classifying drug craving levels
Source: Front Public Health. 2026 May 11;14:1752380. doi: 10.3389/fpubh.2026.1752380 (PMC13199168; doi:10.3389/fpubh.2026.1752380)
Supplement: Supplementary file 4 [file Table_1.docx]

***Supplementary Material***

### **Supplementary Table 1. Summary of the General Information Questionnaire and the Drug Craving Scale**

Name Initials: _______________ Age: _________

Part I. General Information Scale

1. Date of Admission: ____ Year _____ Month _____ Day

2. Gender: ①Male ②Female

3. Date of Birth: ____ Year ____ Month ____ Day

4. Your Education Level: ①Illiterate ②Primary school or below ③Junior high school ④High school or technical secondary school ⑤College ⑥University or above

5. Your Marital Status: ①Unmarried ②Cohabiting ③Married ④Divorced or Widowed

6. Your Occupation before admission: ①Professional/Technical personnel (e.g., doctor, teacher, engineer) ②Service worker ③Businessperson/Merchant ④General laborer/Worker ⑤Agricultural laborer (e.g., farmer, forestry worker) ⑥Temporary worker/Casual laborer ⑦Unemployed ⑧Other (Please specify: ___________ )

7. Monthly household income per capita: ________ Yuan

8. Family atmosphere: ① Harmonious ② Average ③ Tense

9. Types of drugs you used: (Multiple choices allowed) ①Methamphetamine ②MDMA ③Amphetamines④Ketamine ⑤Heroin ⑥Other ___________

10. What was the primary method of drug use in the past? ①Snorting/Smoking ②Injection ③Oral ingestion ④Other ___________

11. Your years of drug use: _______ years

12. When you were using drugs, the average daily amount used was: _________ grams/day

13. Expected Release Date: ____ Year ____ Month ____ Day

Part II. Craving Scale

Please fill this out based on your true feelings and experiences over the past month. Mark a "√" next to the number that best applies to you for each statement. The numbers mean: 1=Strongly Disagree; 2=Disagree; 3=Slightly Disagree; 4=Neutral; 5=Slightly Agree; 6=Agree; 7=Strongly Agree

1. I often think about the pleasant feelings of using drugs.......................…1 2 3 4 5 6 7

2. If I have insomnia, I think about using drugs...................................……1 2 3 4 5 6 7

3.Thinking about places where I bought drugs makes me want to use……1 2 3 4 5 6 7

4. If I don't have a job after release, I might relapse................................…1 2 3 4 5 6 7

5. Recalling the euphoric state from drug use makes me crave it…………1 2 3 4 5 6 7

6. If I have a headache or feel unwell, I think about using drugs……….....1 2 3 4 5 6 7

7. I often think about the person who sold me drugs...................................1 2 3 4 5 6 7

8. If someone offered me drugs, I wouldn't refuse.......................................1 2 3 4 5 6 7

9. I often crave drugs and feel unable to control it.......................................1 2 3 4 5 6 7

10. If I have a poor appetite, I think about using drugs................................1 2 3 4 5 6 7

11. When I feel irritable and restless, I think about using drugs..................1 2 3 4 5 6 7

12. I think about the places where I normally used drugs............................1 2 3 4 5 6 7

13. If I had a lot of money, I would think about buying drugs.....................1 2 3 4 5 6 7

14. I often recall the euphoria from drug use...............................................1 2 3 4 5 6 7

15. If I were extremely angry, it would be great to have drugs....................1 2 3 4 5 6 7

16. If I feel particularly depressed, using drugs would solve it....................1 2 3 4 5 6 7

17. If I feel physically weak, using drugs should make me feel better........1 2 3 4 5 6 7

18. If I feel wronged, I think about using drugs...........................................1 2 3 4 5 6 7

19. Thinking about the physical form of drugs makes me want to use them..............................................................................................................1 2 3 4 5 6 7

20. When talking about the feelings of drug use, I want to use immediately..................................................................................................1 2 3 4 5 6 7

21. If drug-using friends persuade me after release, I'd want to use immediately..................................................................................................1 2 3 4 5 6 7

22. When feeling very pleased with myself, it would be even better to have a fix.................................................................................................................1 2 3 4 5 6 7

23. When I'm enduring frustration, using drugs would make it okay..........1 2 3 4 5 6 7

24. If I see others using drugs, I would also really want to use...................1 2 3 4 5 6 7

25. If I am not responsible towards my family, I might relapse...................1 2 3 4 5 6 7

26. I often recall the "high" or intoxicated feeling from drug use................1 2 3 4 5 6 7

27. When lonely and bored, I naturally think about scenes of using drugs..1 2 3 4 5 6 7

28. I can often recall the appearance, address, and contact details of drug-using friends...........................................................................................................1 2 3 4 5 6 7

29. If relatives are still using drugs, I might use them.................................1 2 3 4 5 6 7

30. I often recall the satisfaction after using drugs.......................................1 2 3 4 5 6 7

31. If my spirits are particularly low, I particularly want to use drugs........1 2 3 4 5 6 7

32. If I experience failure in marriage or romance, I might relapse.............1 2 3 4 5 6 7

33. I recall the feeling of relaxation after using drugs..................................1 2 3 4 5 6 7

34. When I feel pessimistic and hopeless, I crave drugs..............................1 2 3 4 5 6 7

### **Supplementary Table 2. Detailed performance metrics of 10-fold cross-validation for seven machine learning models**

| **Model** | **Fold** | **Accuracy** | **Recall(weighted)** | **Precision(weighted)** | **F1-score (Weighted)** | **AUC (One-vs-Rest)** | **Matthews Correlation Coefficient** | **Negative Log Loss** |
| --- | --- | --- | --- | --- | --- | --- | --- | --- |
| Logistic Regression | 1 | 0.7952 | 0.7952 | 0.7818 | 0.7820 | 0.9026 | 0.4623 | -0.4343 |
|  | 2 | 0.8434 | 0.8434 | 0.8237 | 0.8269 | 0.9002 | 0.5757 | -0.4156 |
|  | 3 | 0.8434 | 0.8434 | 0.8032 | 0.8228 | 0.9183 | 0.5708 | -0.3710 |
|  | 4 | 0.8434 | 0.8434 | 0.8354 | 0.8342 | 0.8787 | 0.5605 | -0.4358 |
|  | 5 | 0.7952 | 0.7952 | 0.7252 | 0.7555 | 0.9259 | 0.3778 | -0.3944 |
|  | 6 | 0.8675 | 0.8675 | 0.8738 | 0.8626 | 0.9530 | 0.6438 | -0.3015 |
|  | 7 | 0.8313 | 0.8313 | 0.7743 | 0.8012 | 0.8574 | 0.5151 | -0.5167 |
|  | 8 | 0.8049 | 0.8049 | 0.8041 | 0.7960 | 0.9355 | 0.5490 | -0.4536 |
|  | 9 | 0.8049 | 0.8049 | 0.7905 | 0.7934 | 0.8948 | 0.4694 | -0.4763 |
|  | 10 | 0.8171 | 0.8171 | 0.8072 | 0.8079 | 0.9131 | 0.5113 | -0.4356 |
| XGBoost | 1 | 0.8193 | 0.8193 | 0.8309 | 0.8238 | 0.9185 | 0.5676 | -0.6010 |
|  | 2 | 0.8193 | 0.8193 | 0.8135 | 0.8157 | 0.9082 | 0.5366 | -0.6089 |
|  | 3 | 0.8554 | 0.8554 | 0.8510 | 0.8474 | 0.9114 | 0.6144 | -0.4995 |
|  | 4 | 0.7952 | 0.7952 | 0.7670 | 0.7777 | 0.8816 | 0.4263 | -0.6456 |
|  | 5 | 0.7952 | 0.7952 | 0.7963 | 0.7873 | 0.8641 | 0.4279 | -0.6849 |
|  | 6 | 0.8554 | 0.8554 | 0.8415 | 0.8411 | 0.9313 | 0.6030 | -0.3947 |
|  | 7 | 0.8675 | 0.8675 | 0.8593 | 0.8608 | 0.8760 | 0.6390 | -0.5360 |
|  | 8 | 0.7927 | 0.7927 | 0.8288 | 0.8031 | 0.9081 | 0.5256 | -0.6512 |
|  | 9 | 0.8049 | 0.8049 | 0.7864 | 0.7936 | 0.9221 | 0.4648 | -0.6008 |
|  | 10 | 0.8659 | 0.8659 | 0.8623 | 0.8639 | 0.9460 | 0.6512 | -0.3408 |
| LightGBM | 1 | 0.8193 | 0.8193 | 0.8186 | 0.8173 | 0.8857 | 0.5507 | -0.8001 |
|  | 2 | 0.8072 | 0.8072 | 0.7888 | 0.7956 | 0.9151 | 0.5025 | -0.6643 |
|  | 3 | 0.8675 | 0.8675 | 0.8632 | 0.8625 | 0.9026 | 0.6562 | -0.6539 |
|  | 4 | 0.8072 | 0.8072 | 0.782 | 0.7916 | 0.8437 | 0.4581 | -0.9578 |
|  | 5 | 0.7711 | 0.7711 | 0.7833 | 0.7607 | 0.8292 | 0.3687 | -0.9779 |
|  | 6 | 0.8554 | 0.8554 | 0.8512 | 0.8491 | 0.9152 | 0.6002 | -0.6159 |
|  | 7 | 0.8434 | 0.8434 | 0.8416 | 0.8403 | 0.8877 | 0.5862 | -0.7706 |
|  | 8 | 0.7561 | 0.7561 | 0.7866 | 0.7652 | 0.8882 | 0.4291 | -1.0140 |
|  | 9 | 0.8293 | 0.8293 | 0.8115 | 0.8148 | 0.9442 | 0.5210 | -0.6962 |
|  | 10 | 0.8780 | 0.8780 | 0.8788 | 0.8778 | 0.9560 | 0.6873 | -0.3715 |
| Decision Tree | 1 | 0.7952 | 0.7952 | 0.8138 | 0.8001 | 0.8046 | 0.5211 | -5.7352 |
|  | 2 | 0.7470 | 0.7470 | 0.7555 | 0.7507 | 0.8233 | 0.3969 | -6.6107 |
|  | 3 | 0.8675 | 0.8675 | 0.8616 | 0.8611 | 0.8582 | 0.6444 | -3.9570 |
|  | 4 | 0.8193 | 0.8193 | 0.8095 | 0.8138 | 0.8036 | 0.5155 | -4.8465 |
|  | 5 | 0.8072 | 0.8072 | 0.8393 | 0.8039 | 0.7230 | 0.5182 | -6.5550 |
|  | 6 | 0.7590 | 0.759 | 0.7452 | 0.7517 | 0.7115 | 0.3533 | -7.0213 |
|  | 7 | 0.8554 | 0.8554 | 0.8616 | 0.8575 | 0.8066 | 0.6330 | -5.2460 |
|  | 8 | 0.7317 | 0.7317 | 0.7285 | 0.7267 | 0.6879 | 0.3148 | -8.8334 |
|  | 9 | 0.7683 | 0.7683 | 0.7446 | 0.7545 | 0.7882 | 0.3657 | -6.2299 |
|  | 10 | 0.8537 | 0.8537 | 0.8541 | 0.8514 | 0.8574 | 0.6240 | -3.5870 |
| Gradient Boosting | 1 | 0.7952 | 0.7952 | 0.7964 | 0.7944 | 0.9083 | 0.4997 | -0.4791 |
|  | 2 | 0.8313 | 0.8313 | 0.8313 | 0.8313 | 0.8956 | 0.5826 | -0.4569 |
|  | 3 | 0.8916 | 0.8916 | 0.8982 | 0.8803 | 0.8930 | 0.7150 | -0.4126 |
|  | 4 | 0.8554 | 0.8554 | 0.838 | 0.8382 | 0.8627 | 0.5909 | -0.4601 |
|  | 5 | 0.8193 | 0.8193 | 0.8147 | 0.8067 | 0.9157 | 0.4815 | -0.4490 |
|  | 6 | 0.9157 | 0.9157 | 0.9152 | 0.9089 | 0.9610 | 0.7756 | -0.2604 |
|  | 7 | 0.8675 | 0.8675 | 0.8708 | 0.861 | 0.8908 | 0.6382 | -0.3996 |
|  | 8 | 0.7683 | 0.7683 | 0.7935 | 0.7771 | 0.8912 | 0.4516 | -0.5467 |
|  | 9 | 0.8415 | 0.8415 | 0.8242 | 0.8269 | 0.9120 | 0.5544 | -0.4210 |
|  | 10 | 0.8902 | 0.8902 | 0.8986 | 0.8926 | 0.9479 | 0.7303 | -0.3177 |
| GaussianNB | 1 | 0.7349 | 0.7349 | 0.7702 | 0.7490 | 0.7936 | 0.4118 | -0.7842 |
|  | 2 | 0.7108 | 0.7108 | 0.7298 | 0.7154 | 0.7820 | 0.2909 | -0.7879 |
|  | 3 | 0.7711 | 0.7711 | 0.7666 | 0.7684 | 0.8240 | 0.3965 | -0.6008 |
|  | 4 | 0.6386 | 0.6386 | 0.6513 | 0.6444 | 0.6763 | 0.0806 | -1.0155 |
|  | 5 | 0.6747 | 0.6747 | 0.7235 | 0.6928 | 0.7334 | 0.2735 | -1.1911 |
|  | 6 | 0.8072 | 0.8072 | 0.8260 | 0.8144 | 0.8906 | 0.5400 | -0.9060 |
|  | 7 | 0.6867 | 0.6867 | 0.7020 | 0.6897 | 0.8142 | 0.1981 | -1.0929 |
|  | 8 | 0.7195 | 0.7195 | 0.7487 | 0.7317 | 0.7708 | 0.3498 | -0.9267 |
|  | 9 | 0.6585 | 0.6585 | 0.6793 | 0.6679 | 0.7283 | 0.1694 | -0.8258 |
|  | 10 | 0.6341 | 0.6341 | 0.7014 | 0.6618 | 0.7487 | 0.2055 | -0.9997 |
| KNN | 1 | 0.6867 | 0.6867 | 0.5530 | 0.6126 | 0.5341 | -0.0829 | -3.9551 |
|  | 2 | 0.7349 | 0.7349 | 0.6373 | 0.6657 | 0.5114 | 0.1031 | -5.5364 |
|  | 3 | 0.7229 | 0.7229 | 0.6103 | 0.6533 | 0.5389 | -0.0062 | -4.7601 |
|  | 4 | 0.7349 | 0.7349 | 0.6217 | 0.6605 | 0.5298 | 0.0200 | -3.4930 |
|  | 5 | 0.7711 | 0.7711 | 0.7639 | 0.6822 | 0.5571 | 0.1980 | -4.2629 |
|  | 6 | 0.759 | 0.759 | 0.6765 | 0.6902 | 0.6148 | 0.1478 | -3.0012 |
|  | 7 | 0.7349 | 0.7349 | 0.6495 | 0.6788 | 0.4847 | 0.1062 | -3.9693 |
|  | 8 | 0.7683 | 0.7683 | 0.6993 | 0.7178 | 0.5836 | 0.2473 | -3.5073 |
|  | 9 | 0.7561 | 0.7561 | 0.5717 | 0.6511 | 0.5957 | 0.0000 | -4.3066 |
|  | 10 | 0.7317 | 0.7317 | 0.5671 | 0.639 | 0.5471 | -0.0763 | -4.3192 |

### **Supplementary Table 3. Reliability Test Results for the Drug Craving Scale**

| Item | Raw Cronbach's α | Standardized Cronbach's α | Guttman's λ6 (SMC) | Average r | Signal-to-Noise Ratio (S/N) | SE(α) | Variance of r | Median r |
| --- | --- | --- | --- | --- | --- | --- | --- | --- |
| 1 | 0.9799 | 0.9813 | 0.9849 | 0.6067 | 52.4505 | 0.0009 | 0.0043 | 0.6066 |
| 2 | 0.9792 | 0.9807 | 0.9845 | 0.5988 | 50.7431 | 0.0009 | 0.0057 | 0.6018 |
| 3 | 0.9793 | 0.9807 | 0.9845 | 0.5990 | 50.7937 | 0.0009 | 0.0057 | 0.6044 |
| 4 | 0.9793 | 0.9807 | 0.9846 | 0.5994 | 50.8800 | 0.0009 | 0.0057 | 0.6044 |
| 5 | 0.9794 | 0.9809 | 0.9847 | 0.6013 | 51.2713 | 0.0009 | 0.0055 | 0.6062 |
| 6 | 0.9793 | 0.9807 | 0.9846 | 0.5997 | 50.9383 | 0.0009 | 0.0056 | 0.6044 |
| 7 | 0.9794 | 0.9808 | 0.9847 | 0.6007 | 51.1538 | 0.0009 | 0.0055 | 0.6055 |
| 8 | 0.9794 | 0.9808 | 0.9847 | 0.6002 | 51.0323 | 0.0009 | 0.0057 | 0.6064 |
| 9 | 0.9792 | 0.9807 | 0.9844 | 0.5986 | 50.7020 | 0.0009 | 0.0053 | 0.6014 |
| 10 | 0.9792 | 0.9807 | 0.9845 | 0.5988 | 50.7536 | 0.0009 | 0.0056 | 0.6018 |
| 11 | 0.9793 | 0.9807 | 0.9846 | 0.5996 | 50.9074 | 0.0009 | 0.0056 | 0.6034 |
| 12 | 0.9793 | 0.9808 | 0.9847 | 0.6000 | 51.0041 | 0.0009 | 0.0057 | 0.6044 |
| 13 | 0.9791 | 0.9806 | 0.9845 | 0.5973 | 50.4326 | 0.0009 | 0.0057 | 0.6003 |
| 14 | 0.9790 | 0.9804 | 0.9844 | 0.5960 | 50.1499 | 0.0010 | 0.0055 | 0.5986 |
| 15 | 0.9801 | 0.9812 | 0.9851 | 0.6053 | 52.1308 | 0.0009 | 0.0049 | 0.6071 |
| 16 | 0.9791 | 0.9806 | 0.9845 | 0.5973 | 50.4207 | 0.0009 | 0.0057 | 0.5992 |
| 17 | 0.9792 | 0.9807 | 0.9846 | 0.5986 | 50.6954 | 0.0009 | 0.0057 | 0.6028 |
| 18 | 0.9791 | 0.9805 | 0.9845 | 0.5967 | 50.3129 | 0.0009 | 0.0056 | 0.5992 |
| 19 | 0.9790 | 0.9804 | 0.9844 | 0.5959 | 50.1438 | 0.0010 | 0.0054 | 0.5992 |
| 20 | 0.9793 | 0.9806 | 0.9846 | 0.5983 | 50.6416 | 0.0009 | 0.0056 | 0.6006 |
| 21 | 0.9791 | 0.9806 | 0.9845 | 0.5972 | 50.4162 | 0.0009 | 0.0055 | 0.5994 |
| 22 | 0.9791 | 0.9805 | 0.9844 | 0.5962 | 50.2067 | 0.0009 | 0.0056 | 0.5986 |
| 23 | 0.9791 | 0.9805 | 0.9845 | 0.5970 | 50.3692 | 0.0009 | 0.0057 | 0.5994 |
| 24 | 0.9791 | 0.9806 | 0.9845 | 0.5975 | 50.4668 | 0.0009 | 0.0056 | 0.5995 |
| 25 | 0.9791 | 0.9806 | 0.9845 | 0.5973 | 50.4376 | 0.0009 | 0.0054 | 0.6006 |
| 26 | 0.9790 | 0.9804 | 0.9843 | 0.5959 | 50.1393 | 0.0010 | 0.0054 | 0.6003 |
| 27 | 0.9791 | 0.9806 | 0.9845 | 0.5973 | 50.4366 | 0.0009 | 0.0056 | 0.6003 |
| 28 | 0.9792 | 0.9806 | 0.9845 | 0.5982 | 50.615 | 0.0009 | 0.0057 | 0.6017 |
| 29 | 0.9791 | 0.9805 | 0.9844 | 0.5965 | 50.2555 | 0.0009 | 0.0055 | 0.5995 |
| 30 | 0.9790 | 0.9805 | 0.9843 | 0.5964 | 50.2501 | 0.0009 | 0.0053 | 0.6003 |
| 31 | 0.9792 | 0.9806 | 0.9845 | 0.5973 | 50.4347 | 0.0009 | 0.0057 | 0.5992 |
| 32 | 0.9792 | 0.9806 | 0.9844 | 0.5978 | 50.5276 | 0.0009 | 0.0054 | 0.6014 |
| 33 | 0.9792 | 0.9806 | 0.9843 | 0.5976 | 50.4876 | 0.0009 | 0.0053 | 0.6014 |
| 34 | 0.9791 | 0.9805 | 0.9844 | 0.5967 | 50.3148 | 0.0009 | 0.0056 | 0.5994 |
| result | 0.9796 | 0.9802 | 0.9843 | 0.5931 | 49.5503 | 0.0009 | 0.0052 | 0.5968 |

**Supplementary Figure 1. ROC curves comparing the performance of six machine learning models**

**Supplementary Figure 2. Confusion matrices comparing the performance of six machine learning models**

**Supplementary Figure 3. Complete SHAP dependence plot of the logistic regression model**
